# Evaluation of Reference Genes to Analyze Gene Expression in Silverside *Odontesthes humensis* Under Different Environmental Conditions

**DOI:** 10.3389/fgene.2018.00075

**Published:** 2018-03-14

**Authors:** Tony L. R. Silveira, William B. Domingues, Mariana H. Remião, Lucas Santos, Bruna Barreto, Ingrid M. Lessa, Antonio Sergio Varela Junior, Diego Martins Pires, Carine Corcini, Tiago Collares, Fabiana K. Seixas, Ricardo B. Robaldo, Vinicius F. Campos

**Affiliations:** ^1^Laboratory of Structural Genomics, Biotechnology Graduate Program, Federal University of Pelotas, Pelotas, Brazil; ^2^Institute of Biological Sciences, Federal University of Rio Grande, Rio Grande, Brazil; ^3^Veterinary Faculty, Federal University of Pelotas, Pelotas, Brazil; ^4^Laboratory of Cancer Biotechnology, Biotechnology Graduate Program, Federal University of Pelotas, Pelotas, Brazil; ^5^Laboratory of Physiology, Institute of Biology, Federal University of Pelotas, Pelotas, Brazil

**Keywords:** algorithm, expression, fish, gene, normalization, real time PCR, sequencing, validation

## Abstract

Some mammalian reference genes, which are widely used to normalize the qRT-PCR, could not be used for this purpose due to its high expression variation. The normalization with false reference genes leads to misinterpretation of results. The silversides (*Odontesthes* spp.) has been used as models for evolutionary, osmoregulatory and environmental pollution studies but, up to now, there are no studies about reference genes in any *Odontesthes* species. Furthermore, many studies on silversides have used reference genes without previous validations. Thus, present study aimed to was to clone and sequence potential reference genes, thereby identifying the best ones in *Odontesthes humensis* considering different tissues, ages and conditions. For this purpose, animals belonging to three ages (adults, juveniles, and immature) were exposed to control, Roundup®, and seawater treatments for 24 h. Blood samples were subjected to flow-cytometry and other collected tissues to RNA extraction; cDNA synthesis; molecular cloning; DNA sequencing; and qRT-PCR. The candidate genes tested included *18s, actb, ef1a, eif3g, gapdh, h3a, atp1a*, and *tuba*. Gene expression results were analyzed using five algorithms that ranked the candidate genes. The flow-cytometry data showed that the environmental challenges could trigger a systemic response in the treated fish. Even during this systemic physiological disorder, the consensus analysis of gene expression revealed *h3a* to be the most stable gene expression when only the treatments were considered. On the other hand, *tuba* was the least stable gene in the control and *gapdh* was the least stable in both Roundup® and seawater groups. In conclusion, the consensus analyses of different tissues, ages, and treatments groups revealed that *h3a* is the most stable gene whereas *gapdh* and *tuba* are the least stable genes, even being considered two constitutive genes.

## Introduction

Northern-blotting, ribonuclease protection assay, microarray, RNA-Seq, semi-quantitative RT-PCR, and quantitative RT-PCR (qRT-PCR) are among the most currently used techniques for evaluation of gene expression levels. The latter stands out for fast readout, high throughput and high automation potential (Tang et al., 2012). In absolute quantification of qRT-PCR, the increasing level of fluorescent signal emitted by the amplified products is compared to a standard curve. The relative quantification describes the change in the expression pattern of genes of interest when subjected to certain controlled situations compared to the constant expression of reference genes (RGs), also known as housekeeping genes under same conditions. Therefore, it is extremely important to have a stable and constant expression pattern of RGs regardless of gender, age, organ or tissue, and even after environmental changes. Thus, it is necessary to validate diverse RGs for various species and their stages of development in different tissues, as well as for each type of controlled environmental condition (Livak and Schmittgen, 2001; Zheng and Sun, 2011).

However, most studies related to the quantification of gene expression in various species of teleosts are not based on RGs displaying unchanged expression. Most studies use RGs properly confirmed in mammalian species to normalize the relative quantification using qRT-PCR of non-mammalian species, such as fishes. The main RGs from mammals that have been empirically used to normalize experiments in teleost species are the *GAPDH* (Lin et al., 2004); *ACTB* (Choi and An, 2008; Nawata et al., 2010); *EF1*α (Scott et al., 2004; Nilsen et al., 2007; Patterson et al., 2012; Sinha et al., 2015); *RPL* (Hsu et al., 2014) and *18S* (Sinha et al., 2015). Studies also report the distribution of RGs in the tissues; however, these genes are not objectively evaluated in relation to the maintenance of the constant expression during development stages of individuals, in both genders or in different controlled environmental conditions (Tomy et al., 2009; Hsu et al., 2014).

Numerous studies have reported that some mammalian RGs, which are widely used to normalize the qRT-PCR reactions of different species (i.e., *ACTB* and *GAPDH*), could not be used owing to their high variation in expression levels (Bustin, 2002; Dheda et al., 2004; Silver et al., 2006). This could lead to misinterpretation of results. It is impossible to find suitable RGs that exhibit constant expression pattern for all species and under all experimental conditions (Zheng and Sun, 2011; Tang et al., 2012). Due to these reasons, the identification of RGs for each species and each experimental condition is justified.

The silversides (*Odontesthes* spp.) comprise a genus of fish that are naturally endemic to the South American waters (Bemvenuti, 2006). This genus consists of the biggest number of species of the South American atherinopsids (Dyer, 2006). In nature, the silversides are usually found in southern Brazil, Uruguay, and Argentina (Bemvenuti, 2006). In these regions, some species have economic importance for production, flesh commercialization and sport fishing (Menone et al., 2000; Somoza et al., 2008). Furthermore, some silversides have been used as experimental models for evolutionary, osmoregulatory, and environmental pollution studies due to the radiation after continental waters invasion from ocean, euryhaline biology and inhabitation of environments contaminated by glyphosate coming from rice and soybean monocultures in fields near to the habitat of silversides (Menone et al., 2000; Carriquiriborde and Ronco, 2006; Tsuzuki et al., 2007; Piedras et al., 2009; Bloom et al., 2013; Hughes et al., 2017; Zebral et al., 2017). Moreover, the silversides have already been utilized to address various aspects of gene expression (Strobl-Mazzulla et al., 2005; Karube et al., 2007; Fernandino et al., 2008, 2011; Majhi et al., 2009; Miranda et al., 2009; Blasco et al., 2010; Gómez-Requeni et al., 2012; Pérez et al., 2012; González et al., 2015). The expression pattern of a gene can be used to assess physiological responses of fishes to environmental changes, such as those to which some silversides are exposed in their habitat (salinity variations and contamination by glyphosate) (Wang et al., 2014; Velasques et al., 2016).

Similarly, *Odontesthes humensis* de Buen, 1953, a native species to coastal lagoons from southern Brazil, Uruguay and Argentina, has similar potential to be used as a biological model. However, a previous basic knowledge about the RGs is necessary to employ modern techniques such as qRT-PCR to study the gene expression of different genes of interest, independent from the biological model. Up to now, no studies have focused on the sequence determination and validation of RGs in any species of *Odontesthes* spp. Due to this, the present study aimed to clone and sequence eight potential RGs, thereby identifying and validating the best RGs in *O. humensis* using different tissues, stages of development and a natural and an anthropized environmental conditions, to standardize the qRT-PCR technique.

## Materials and methods

### Animals and sample collection

The silversides *Odontesthes humensis* used in this study was procreated from the eggs previously collected in nature (Arroio Grande, Brazil: 32°14′15″S/53°05′13″W) and hatched in tanks. The silversides were kept within an experimental room in nine 1,000 L circular plastic tanks with water at a temperature of 13.3 ± 1.8°C, pH 7.0 ± 0.7, salinity 3.2 ± 0.67 ppt, dissolved oxygen 9.5 ± 0.5 mg/L, ammonia levels lower than 0.6 mg/L and a natural photoperiod of winter. The tank sides were opaque to reduce visual stress, and once a week 2/3 of the water in the tanks were renewed. Three tanks were occupied by 3-year-old fish (5 animals/tank), classified as adults considering the complete development of gonads. Other three tanks were occupied by 1.5-year-old fish (5 animals/tank), classified as juveniles considering the partial development of gonads. In addition, three tanks were occupied by 6-month-old silversides (5 animals/tank), classified as immature due to the undifferentiated gonads. The animals were fed thrice a day on a commercial diet (Supra, 38% crude protein) and zooplankton *ad libitum*. The acclimation period was 4 weeks under these conditions.

The experimental design consisted of three treatment groups: the “control group,” exposed to water in previously mentioned conditions; the “Roundup® group,” exposed to Roundup® Transorb (a glyphosate based herbicide) diluted in water at a concentration of 10 mg.L^−1^ (acid equivalent, a.e.) of glyphosate; and the “seawater group,” exposed to seawater at 30 ppt (dissolution of non-iodized sea salt in the medium). Three tanks were prepared for each treatment, the first one received the adults, the second one received the juveniles, and the third one received the immature individuals. After the acclimation period, the animals were exposed to treatments for 24 h. The water quality parameters did not differ statistically from those observed in the acclimatization period, except for the salinity of the group exposed to seawater. During the experimental period, the fish continued to be fed as during the acclimation period.

Each animal was hooked from the tank and anesthetized by submersion in benzocaine at 50 ppm. Three fish from each tank were collected, leading to a total of 27 animals. The animals were weighed, measured, and euthanized by cranial spinal section and excision of the brain. Adults, juveniles and immature silversides had mean lengths of 20.2 ± 2.3 cm, 15.2 ± 1.2 cm, and 11.7 ± 0.7 cm and mean weight of 55.2 ± 11.7 g, 24.6 ± 6.5 g, and 9.6 ± 1.9 g, respectively. Brain, hepatopancreas, gills, and kidney (the main organs affected by glyphosate and/or hyperosmotic environments in fishes; Tipsmark et al., 2004; Carriquiriborde and Ronco, 2006; Hiroi and McCormick, 2012; Harayashiki et al., 2013; Velasques et al., 2016) from adults, juveniles and immature silversides in the three treated groups were collected and preserved in liquid nitrogen (N_2_). Furthermore, a total of 20 μL of peripheral blood was collected by puncturing the caudal fin. Each blood sample was added to 1 mL of fetal bovine serum (FBS), stored at 4°C, and protected from the light until used for flow-cytometry analysis. The animal use and all handling practices were approved by the Ethics Committee on Animal Experimentation of the Federal University of Pelotas (process no. 23110.007018/2015-85). The Roundup® residual from experimental tanks was sent to university chemical waste facility to to be treated prior to disposal.

### Flow-cytometric analysis

The flow-cytometry analysis was performed using the Attune® Acoustic Focusing Flow Cytometer (Applied Biosystems, USA) to evaluate the efficacy of treatments to affect the physiology of silversides. Each blood sample was exposed to 2 mM of Hoechst 33342 (H33342; Sigma-Aldrich, USA) for 5 min before each read, except in the DNA damage analysis. Events were detected by fluorochrome with violet laser (405 nm) and photomultiplier (PMT) VL1 (filter 450/40 nm). The green, orange, and red fluorescences were analyzed by blue laser (488 nm) and PMTs BL1 (filter 530/30 nm), BL2 (filter 575/24 nm), and BL3 (filter > 640 nm) filters, respectively. Cytometry fluorescence stability was tested daily using standard beads (Invitrogen, USA). The acquisition rate was 200 events/s totaling 20,000 events per sample. All assays were performed in triplicate. The results were analyzed using the Attune Cytometric Software v2.1. The non-cellular events were eliminated from the analysis by scatter plots of forward scatter (FSC) × side scatter (SSC) (Petrunkina et al., 2005) and negative fluorescence of H33342 events (debris).

For evaluation of reactive oxygen species (ROS) produced by the erythrocytes, 10 μL of blood sample previously collected and stored was added to 20 μL of saline solution with 2 μM of 2′,7′-dichlorofluorescein diacetate (DCFH-DA) and 5 μM of propidium iodide (PI) fluorescent probes (Sigma-Aldrich Co., USA). The samples were analyzed in triplicate after incubation for 60 min at 22°C in the dark. The ROS production was measured by the median of the intensity of the green fluorescence. Only intact cells (PI negative) were evaluated.

To evaluate DNA damage in the erythrocytes, 10 μL of blood sample previously collected and stored was added to 5 μL of TNE (0.01 M Tris-HCl. 0.15 M NaCl, 0.001 M EDTA, pH 7.2) and 30 s later to 10 μL of Triton 1X (Triton X-100, 1%, v/v). Then, 50 μL acridine orange dye (2 mg/mL, #A6014, Sigma-Aldrich, USA) was added to the sample, followed by incubation from 30 s up to 2 min before each reading. The samples were analyzed in triplicate. The DNA of the erythrocytes was classified as integrated (green fluorescence emission) and damaged (orange/red fluorescence emission). The percentage of DNA fragmentation index (DFI) was obtained by the median of the red fluorescence intensity/(median of the red + green fluorescence intensities) × 100.

### RNA extraction and cDNA synthesis

The total RNA was extracted from tissue samples using the commercial kit RNeasy® Mini Kit (Qiagen, USA) following the manufacturer's instructions. The RNA was treated with DNA-free® Kit (Ambion, USA) to remove genomic DNA contamination. Subsequently, the RNA concentration and purity were measured using a spectrophotometer NanoVue™ Plus (GE Healthcare Life Science, USA), and only the samples presenting high purity (OD_260/280_ ≥ 2.0 nm) were used. In addition, RNA quality was analyzed on the 4,200 TapeStation (Agilent Technologies, USA) system using the TapeStation analysis application. The mean RIN number for each experimental group is listed in Supplementary Table 1. First-strand cDNA synthesis was performed with 2 μg of RNA using the commercial kit High Capacity cDNA Reverse Transcription (Applied Biosystems, USA) according to manufacturer's recommendation. The reactions were run in SimpliAmp™ thermal cycler (Applied Biosystems, USA). Finally, the cDNA was stored at −20°C until its use.

### Amplification and cloning of candidate RGs

The tested candidates RGs were: 18S ribosomal RNA (*18s*), β-actin (*actb*), elongation factor 1 α (*ef1a*), eukaryotic translation initiation factor 3g (*eif3g*), glyceraldehyde-3-phosphate dehydrogenase (*gapdh*), histone h3a (*h3a*), Na^+^/K^+^-ATPase α (*atp1a*), and tubulin-α (*tuba*) (Table 1). The *atp1a* gene represented the control, which is a constitutive gene that is reported to be affected by glyphosate-based herbicides and salt treatments (Shiogiri et al., 2012; Armesto et al., 2014).

**Table 1:** Summary of candidate reference genes evaluated in the present study.

**Gene symbol**	**Gene name**	**Function**	**GenBank accession No**.
*18s*	18S ribosomal RNA	Ribosomal subunit	KU639718
*actb*	β-Actin	Cytoskeletal protein	KX060039
*ef1a*	Elongation factor 1-α	Factor for protein translation	KU639717
*eif3g*	Eukaryotic translation initiation factor 3g	Initiation of protein synthesis	KX060035
*gapdh*	Glyceraldehyde-3-phosphate dehydrogenase	Glycolytic enzyme	KX060038
*h3a*	Histone h3a	Component of nucleosomes	KX060037
*atp1a*	Na^+^/K^+^-ATPase-α	Active transport of Na^+^ and K^+^	KR920364
*tuba*	Tubulin-α	Cytoskeletal protein	KX035015

The gene fragments were amplified by PCR using primers designed in the PriFi online tool (https://services.birc.au.dk/prifi/) after alignments (Supplementary Table 2) of known sequences deposited in the GenBank (Table 2). The PCR was performed using the mix buffer GoTaq® G2 Flexi DNA Polymerase (Promega, USA) following manufacturer's instructions. The reactions were run in the SimpliAmp™ thermal cycler (Applied Biosystems, USA). The PCR parameters were: an initial denaturation step for 1 min at 94°C, followed by 35 cycles at 94°C for 30 s, 59.9 to 65.5°C (depending on the primer sequence, Table 2) for 30 s; and 72°C for 1 min, with a final extension of 5 min at 72°C. To confirm the amplification of the fragments, the reactions were analyzed using 1.5% agarose gel electrophoresis. The PCR products were inserted into the cloning vector pCR™4-TOPO® TA (Invitrogen, USA). The transformed vector was used to transform electrocompetent *Escherichia coli* DH5α.

**Table 2:** Primers information of the candidate reference genes in *Odontesthes humensis*.

**Gene symbol**	**Sequence 5′ → 3′**	**Average T_m_ (°C)**	**Product size (bp)**	**E (%)**	**R^2^**	**Use**
*18s*	GCCGGTACAGTGAAACTGCGAATG	62	716	−	−	Cloning
	TTTCAAAGTAAACGCTTCGGACCCCG					
	AAACGGCTACCACATCCAAG	60	112	100.1	0.998	qRT-PCR
	CAATTACAGGGCCTCGAAAG					
*actb*	AGGAGCACCCWGTCCTGCT	64.3	448	−	−	Cloning
	ATGACCTGTCCGTCRGGCAG					
	AGGCTGTGCTGTCCCTGTAT	60	104	108.2	0.993	qRT-PCR
	CAGGGCGTAACCCTCATAGA					
*ef1a*	GAGCGTGAGCGTGGTATCACC	61.5	680	−	−	Cloning
	ACAGACTTCACCTCAGTGGTCAGGTTG					
	CGTTTCGAGGAAATCCAAAA	60	141	104.5	0.999	qRT-PCR
	CTTGAACCAGCCCATCTTGT					
*eif3g*	AAAGAACTGGAAGAARTTTGGCAACTC	59.9	501	−	−	Cloning
	GGYCTGAAGAGCTCCTGCA					
	GTATTTGCAAAGGCGACCAT	60	118	93.9	0.986	qRT-PCR
	GCAGGCTTGTCTTTGTCTCC					
*gapdh*	GTATGACTCCACCCACGGMCG	65.5	400	−	−	Cloning
	GTAGGCGTGRACKGTGGTCATGAG					
	GGTGGTGCCAAGAGAGTCAT	60	124	90.8	0.989	qRT-PCR
	TAGTTGTGCAGGAGGCATTG					
*h3a*	AGGAAGCAGCTGGCCACC	60	285	−	−	Cloning
	GGCGCACAGGTTGGTGTCCTC					
	CGTGGCTCTGAGAGAGATCC	60	108	105.3	0.995	qRT-PCR
	TCGGTCTTGAAGTCCTGAGC					
*atp1a*	AACCCCAGAGATGCCAA	63	1010	−	−	Cloning
	AAGGCACAGAACCACCA					
	ATACCGGGGCAGTAGGAGAG	60	150	114	0.993	qRT-PCR
	CAGTATCGTGGTGGTGCAGT					
*tuba*	ACTCCATCCTGACCACCCACACCACC	69	589	−	−	Cloning
	GTGGTGTTGCTCAGCATGCACAC					
	ATGAGCAGCTTTCGGTGTCT	60	119	101.8	0.983	qRT-PCR
	ATCACCACGGAACAGTAGGC					

*E, efficience; bp, base pair; R^2^, Pearson coefficient of determination; Tm, melting temperature*.

### Purification and sequencing of the fragments of interest

The transformed *E. coli* colonies were selected and cultured in 3 mL of Luria broth (LB) medium containing kanamycin antibiotic. The resistant bacteria were used to isolate the vector and the fragment was purified using the commercial kit Illustra plasmidPrep Mini Spin (GE Healthcare, USA) following the manufacturer's instructions. The purified samples of ten cloning fragments were submitted to sequencing by BigDye® Terminator v3.1 Cycle Sequencing (Applied Biosystems, USA) following the manufacturer's instructions. The reaction mixtures were incubated in a SimpliAmp™ thermal cycler (Applied Biosystems, USA) under the following parameters: 96°C for 1 min, 35 cycles of 96°C for 1 min, 50°C for 5 s, 60°C for 4 min.

One more purification was carried out using the BigDye® XTerminator™ Purification (Applied Biosystems, USA) according to the manufacturer's instructions. Following the purification, the samples were submitted to sequencing using an Applied Biosystems 3,500 Genetic Analyzer® automatic sequencer (Life Technologies, USA). The sequences were analyzed using the Vector NTI and deposited in GenBank®.

### Gene expression analysis by qRT-PCR

The primers used for qRT-PCR were designed using the previous sequence of the cloned fragments and Primer3 online tool (www.bioinfo.ut.ee/primer3-0.4.0/). The primers used in this study are presented in Table 2. The qRT-PCR was run in a Stratagene® Mx3005P™ Real-Time PCR System (Agilent Technologies, USA) using SYBR® Green PCR Master Mix (Applied Biosystems, USA). The amplification conditions were 95°C for 10 min, 40 cycles at 95°C for 15 s, and 60°C for 60 s followed by the conditions needed to calculate the melting curve. All the reactions were performed in triplicate.

### Data analysis

The normality distribution of flow-cytometry data was evaluated by the Shapiro-Wilk test. None of the evaluated parameters presented normal distribution, thus, the data were submitted to the Kruskal-Wallis non-parametric test followed by Dunn's all-pairwise multiple comparisons, with a significance level of 5%. The results of ROS production and DFI were expressed as the mean fluorescent intensity and mean percentage, respectively, ± standard error of the means (SEM).

The cycle threshold (Ct) values were exported from the detection software MxPro™ v.4.1 (Stratagene, Waldbronn, Germany) to Excel files to facilitate the descriptive analysis and the values transformations required by some software. The specific analyses of stability of gene expression were performed using the comparative delta-Ct (dCt) method (Silver et al., 2006) and geNorm (Vandesompele et al., 2002), NormFinder (Andersen et al., 2004), BestKeeper (Pfaffl et al., 2004), and RefFinder (http://150.216.56.64/referencegene.php?type=reference) statistical approaches as previously reported (Yang et al., 2013; Taki et al., 2014). The results were expressed as average of standard deviation (SD) to dCt method; average expression stability value to geNorm and NormFinder; Pearson's correlation coefficient ([r]) to BestKeeper; and geometric mean of ranking values to RefFinder.

## Results

### Cloning and sequencing of RGs

Except for *atp1a* gene, which was previously known (Silveira et al., 2018), all the other seven silverside *O. humensis* candidate RGs analyzed in this study were cloned and partially sequenced for the first time. Furthermore, the new sequences were deposited in the GenBank® (Table 1). The sequence length of *18s* was 640 base pairs (bp). The sequence lengths of *actb*; *ef1a*; *eif3g*; *gapdh*; *h3a*; and *tuba* cloned fragments from *O. humensis* were 350, 638, 435, 325, 222, and 527 bp, respectively, that encoded 116, 212, 144, 108, 73, and 176 amino acids, respectively. The cloned fragments of *tuba* belongs to the open reading frame (ORF) +1, the fragments of *actb* and *eif3g* are part of the ORF +2, and the fragments of *ef1a, gapdh*, and *h3a* compose the ORF +3.

### Flow-cytometry analysis

**Figure 1:**
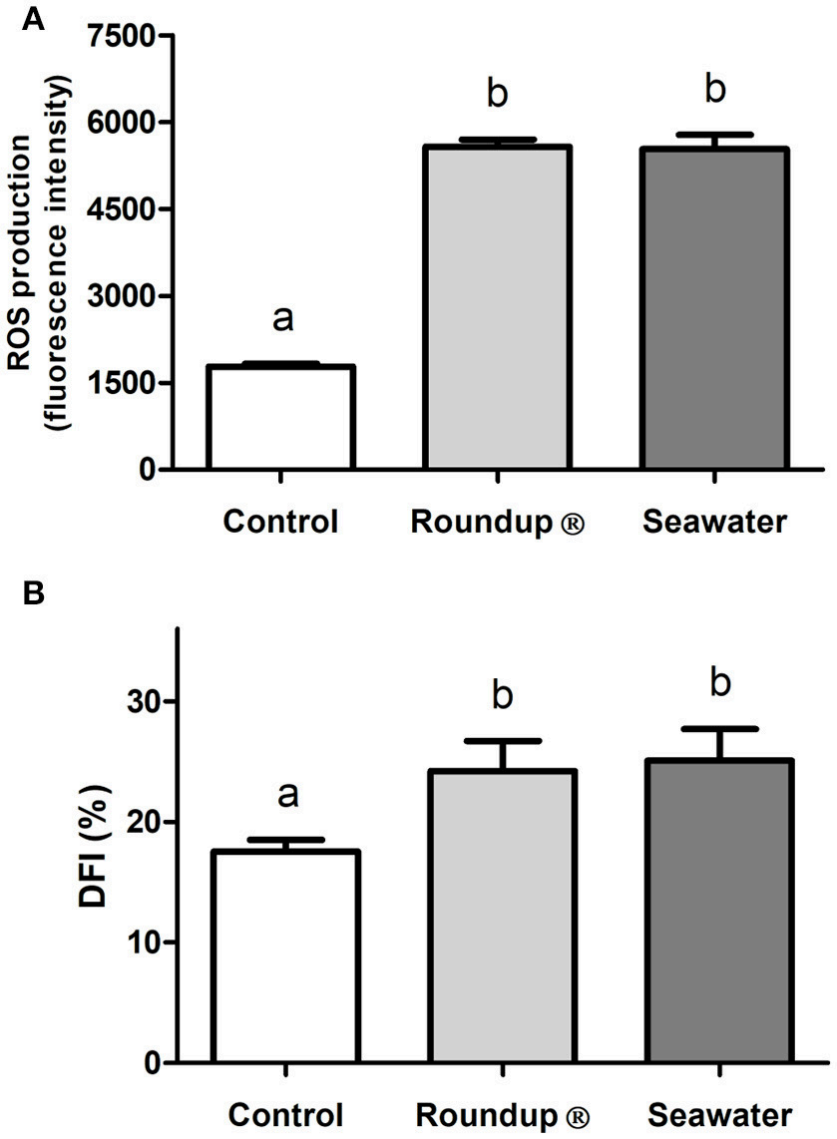
Effects in erythrocytes of *Odontesthes humensis* of exposures to Roundup® and to seawater. Effects of exposures to Roundup® Transorb [10 mg.L^−1^ (acid equivalent) of glyphosate] and to seawater (30 ppt) for 24 h on ROS (Reactive Oxygen Species) production **(A)** and in DFI (DNA Fragmentation Index, %) **(B)**. Mean ± SEM, *n* = 9. Different letters represents significant statistical difference between the measurements of the treatment groups (*P* < 0.05).

The exposure of silversides for 24 h to both Roundup® and seawater caused an increase in the ROS production in erythrocytes (*P* < 0.05) when compared to the control group (Figure 1A). Similarly, the Roundup® and seawater treatments increased (*P* < 0.05) the DFI of the erythrocytes in the exposed silversides (Figure 1B). The increase in ROS production and DNA damage in the treated fish confirmed that both Roundup® and seawater treatments negatively affected the physiology of silversides, thereby generating a systemic effect on fish.

### The comparative dCt method analysis

**Figure 2:**
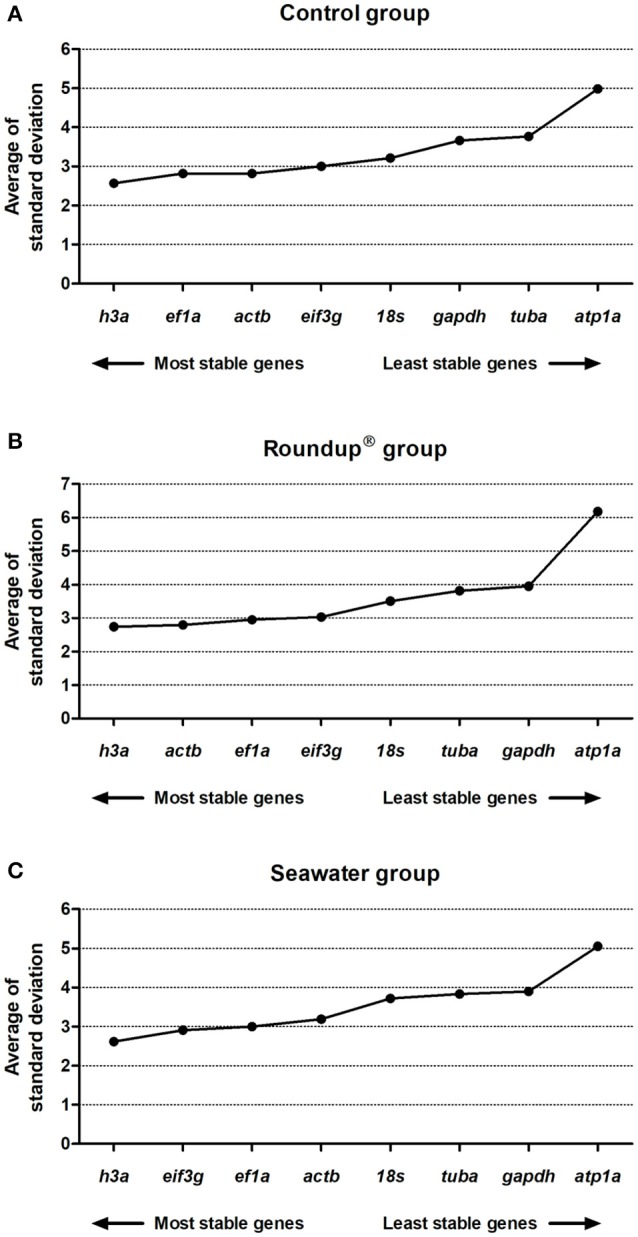
Stability analysis of the candidates reference genes in *Odontesthes humensis* calculated by dCt method. Gene expression stability in silversides exposed to water with quality parameters controlled **(A)**; to Roundup® Transorb [10 mg.L^−1^ (acid equivalent) of glyphosate] **(B)**; and to seawater (30 ppt) **(C)** across the different treatments for 24 h. The most stable genes are displayed on the left, and the least stable genes are displayed on the right of the x-axis.

The comparative dCt method was used to select the most stable RG. A low average of SD value represented a low expression variance, or high stability. The analysis revealed the following order of stability of evaluated genes, from higher to lower, in water with controlled quality parameters (control group): *h3a* (2.57) > *ef1a* (2.82) = *actb* (2.82) > *eif3g* (3.00) > *18s* (3.21) > *gapdh* (3.66) > *tuba* (3.77) > *atp1a* (4.98) (Figure 2A). The ranking order of stability of the evaluated genes upon exposure of silversides to Roundup® treatment was *h3a* (2.74) > *actb* (2.79) > *ef1a* (2.95) > *eif3g* (3.03) > *18s* (3.51) > *tuba* (3.82) > *gapdh* (3.95) > *atp1a* (6.18) (Figure 2B). In saline condition, the seawater treated group presented the following stability order: *h3a* (2.62) > *eif3g* (2.91) > *ef1a* (3.00) > *actb* (3.19) > *18s* (3.72) > *tuba* (3.84) > *gapdh* (3.90) > *atp1a* (5.05) (Figure 2C).

### GeNorm analysis

**Figure 3:**
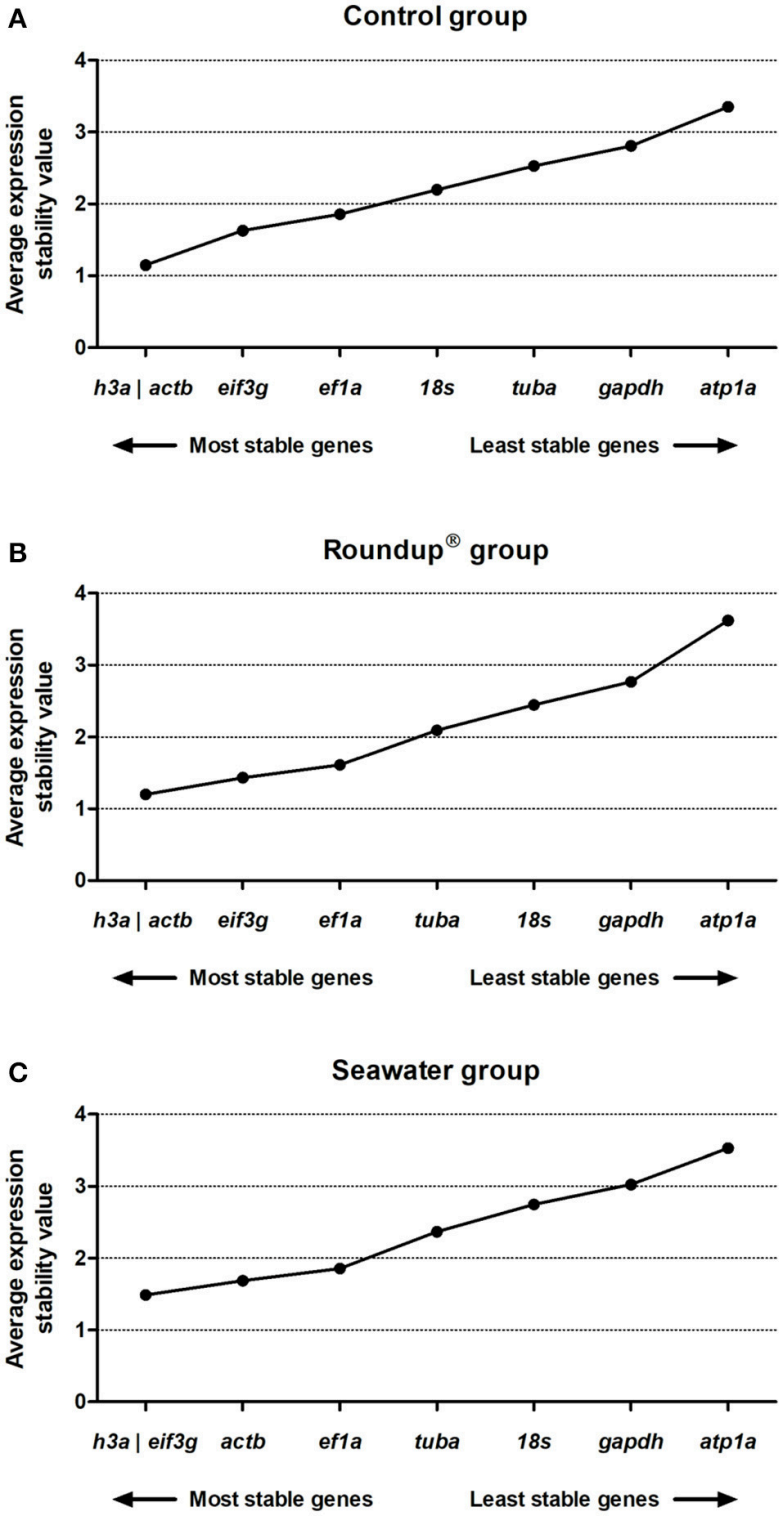
Stability analysis of the candidates reference genes in *Odontesthes humensis* calculated by geNorm algorithm. Gene expression stability in silversides exposed to water with quality parameters controlled **(A)**; to Roundup® Transorb [10 mg.L^−1^ (acid equivalent) of glyphosate] **(B)**; and to seawater (30 ppt) **(C)** across the different treatments for 24 h. The most stable genes are displayed on the left, and the least stable genes are displayed on the right of the x-axis.

The geNorm analysis was carried out for the result data set after transformation of the Ct values into relative quantities through the 2^(minimum Ct value in a set sample−Ct value of a sample)^ formula and compare pairwise variation (SD values) for each gene pair. Then, the geometric mean of SD values was used to calculate the *M-*value. The generation of low average expression stability represents a low variance. The geNorm analysis revealed the following ranking order of stability of genes in the control group: *h3a*/*actb* (1.14) > *eif3g* (1.62) > *ef1a* (1.85) > *18s* (2.19) > *tuba* (2.53) > *gapdh* (2.81) > *atp1a* (3.35) (Figure 3A). In the Roundup® group, the sequence of stability of the evaluated genes from the highest to the lowest was *h3a*/*actb* (1.19) > *eif3g* (1.43) > *ef1a* (1.61) > *tuba* (2.09) > *18s* (2.44) > *gapdh* (2.77) > *atp1a* (3.62) (Figure 3B). When exposed to seawater, the silversides genes exhibited the following stability ranking order: *h3a*/*eif3g* (1.48) > *actb* (1.68) > *ef1a* (1.85) > *tuba* (2.36) > *18s* (2.74) > *gapdh* (3.02) > *atp1a* (3.53) (Figure 3C).

### NormFinder analysis

**Figure 4:**
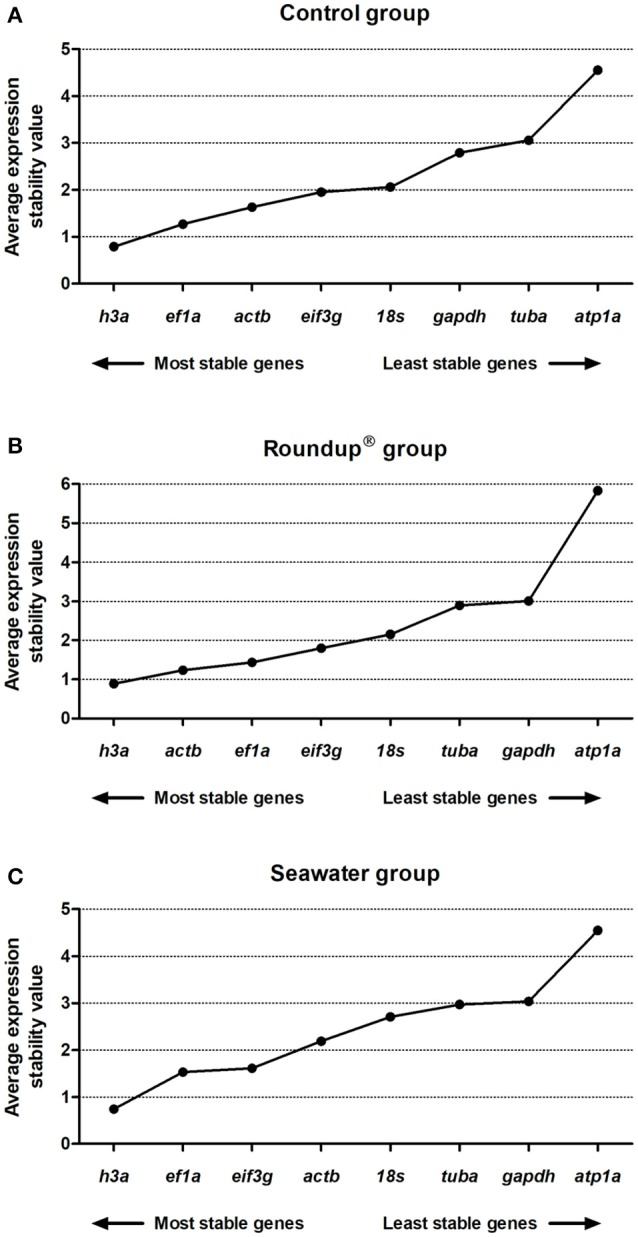
Stability analysis of the candidates reference genes in *Odontesthes humensis* calculated by NormFinder algorithm. Gene expression stability in silversides exposed to water with quality parameters controlled **(A)**; to Roundup® Transorb [10 mg.L^−1^ (acid equivalent) of glyphosate] **(B)**; and to seawater (30 ppt) **(C)** across the different treatments for 24 h. The most stable genes are displayed on the left, and the least stable genes are displayed on the right of the x-axis.

The NormFinder was applied to analyze the most stable evaluated genes. A low average of expression stability represents a low variance. The NormFinder analysis revealed the ranking order of stability in the control group to be *h3a* (0.79) > *ef1a* (1.26) > *actb* (1.63) > *eif3g* (1.95) > *18s* (2.06) > *gapdh* (2.79) > *tuba* (3.05) > *atp1a* (4.55) (Figure 4A). For the Roundup® group, the ranking of expression stability was *h3a* (0.89) > *actb* (1.23) > *ef1a* (1.43) > *eif3g* (1.79) > *18s* (2.15) > *tuba* (2.89) > *gapdh* (3.01) > *atp1a* (5.83) (Figure 4B). When exposed to the seawater, the ranking order of RGs was *h3a* (0.74) > *ef1a* (1.53) > *eif3g* (1.61) > *actb* (2.19) > *18s* (2.70) > *tuba* (2.97) > *gapdh* (3.04) > *atp1a* (4.54) (Figure 4C). In addition, the best combination of genes for the experimental conditions was evaluated. A comparison of the gene expression in silversides of the control and the Roundup® groups, and of the control and the seawater groups revealed the best combination of genes to be *h3a* and *ef1a* with combined stability values of 0.28 and 0.29, respectively. The best suitable combination of two genes for all three tested conditions was also *h3a* and *ef1a* with a combined stability value of 0.26.

### BestKeeper analysis

The BestKeeper analysis provided two-interpretation-ways to rank the gene stability: one based on the samples SD values of Ct and other based on the Pearson's correlation of expression among the genes. Thus, the genes with low SDs and high correlation with the BestKeeper index (indicating high similarity among the expression levels of the RGs) are ranked as the most stable genes. To construct the consensus comprehensive ranking, the SD values were used, which is a more conservative approach. However, to construct the rankings of the most stable genes with BestKeeper, [r] and *P*-values of Pearson's correlation were employed. This way is more sophisticated and statistically robust, as it results in the rankings more similar to the ones obtained using other algorithms.

**Figure 5:**
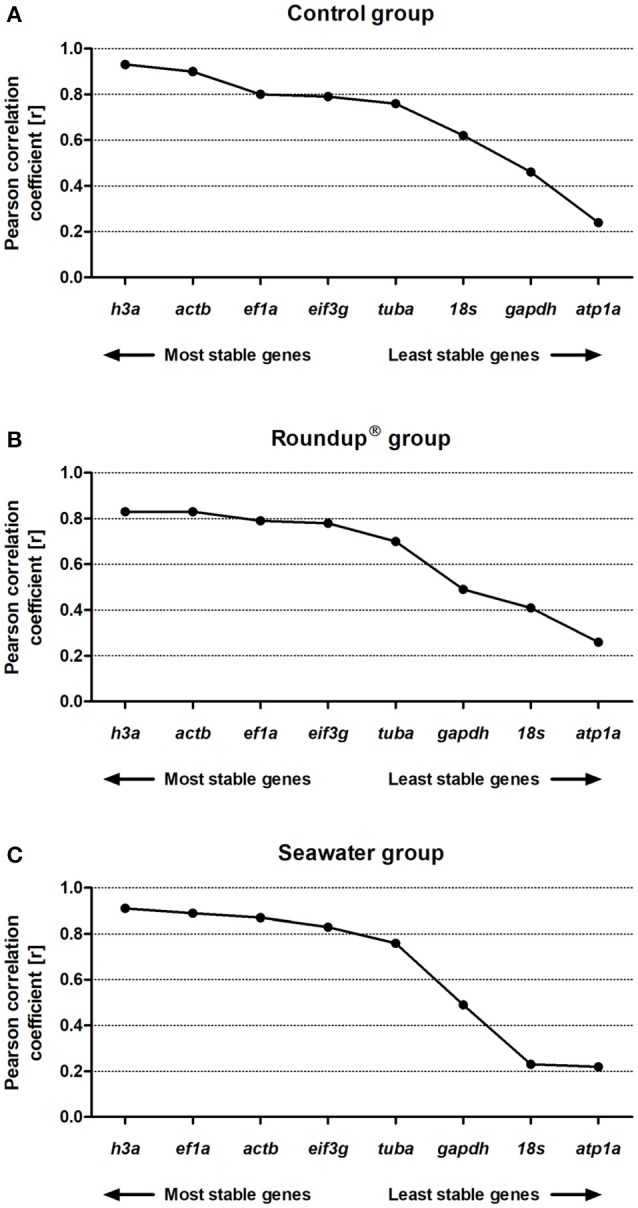
Stability analysis of the candidates reference genes in *Odontesthes humensis* calculated by BestKeeper algorithm. Gene expression stability in silversides exposed to water with quality parameters controlled **(A)**; to Roundup® Transorb [10 mg.L^−1^ (acid equivalent) of glyphosate] **(B)**; and to seawater (30 ppt) **(C)** across the different treatments for 24 h. The most stable genes are displayed on the left, and the least stable genes are displayed on the right of the x-axis.

The analyses based on Pearson's correlation revealed the following ranking order of stability in the control group: *h3a* (0.93) > *actb* (0.90) > *ef1a* (0.80) > *eif3g* (0.79) > *tuba* (0.76) > *18s* (0.62) > *gapdh* (0.46) > *atp1a* (0.24) (Figure 5A). The genes presented a positive correlation with the BestKeeper index with *P* = 0.001 except atp1a with *P* = 0.05. In the Roundup® group the ranking of the best RG was *h3a* (0.83) = *actb* (0.83) > *ef1a* (0.79) > *eif3g* (0.78) > *tuba* (0.70) > *gapdh* (0.49) > *18s* (0.41) > *atp1a* (0.26) (Figure 5B). All correlation analysis presented *P* = 0.001 except *atp1a* with *P* = 0.08. In the seawater group the ranking order from the most to the least stable genes was *h3a* (0.91) > *ef1a* (0.89) > *actb* (0.87) > *eif3g* (0.83) > *tuba* (0.76) > *gapdh* (0.49) > *18s* (0.23) > *atp1a* (0.22) (Figure 5C). The genes *atp1a* and *18s* exhibited *P* = 0.1 and *P* = 0.08, respectively. All the other genes displayed positive correlations with *P* = 0.001.

### RefFinder analysis

The RefFinder is an online tool used to construct a consensus comprehensive ranking of stability of RGs among all the other methods using the calculation of geometric mean for the ranks calculated by each of other algorithms. Candidate genes with the lowest geometric mean are most stable.

Besides the graphical rankings of RefFinder, it presented the consensus results discriminated by treatment group (control, Roundup®, and seawater) by developmental stages (adults, juveniles, and immature) and tissue types (brain, gills, hepatopancreas, and kidney).

In the control group, the most stable gene across the different ages and tissues was *h3a*. The only exception was the brain samples, where the most stable gene was *tuba*. The least stable gene across the developmental stages and tissues was *atp1a* (Table 3).

**Table 3:** Consensus stability ranking by RefFinder of the candidate reference genes in *Odontesthes humensis* under normal conditions.

**Control group**		**Ranking**							
		**1**	**2**	**3**	**4**	**5**	**6**	**7**	**8**
Ages	Adults	*h3a*	*actb*	*18s*	*ef1a*	*eif3g*	*gapdh*	*tuba*	*atp1a*
		1.32	2.00	3.16	3.41	4.68	4.74	6.96	7.74
	Juveniles	*h3a*	*eif3g*	*ef1a*	*18s*	*actb*	*gapdh*	*tuba*	*atp1a*
		1.41	2.45	3.13	3.34	3.66	4.56	7.24	7.74
	Immatures	*h3a*	*18s*	*ef1a*	*actb*	*eif3g*	*gapdh*	*tuba*	*atp1a*
		1.41	2.28	2.51	2.99	4.16	6.48	6.48	8.00
Tissues	Brain	*tuba*	*h3a*	*eif3g*	*18s*	*actb*	*ef1a*	*gapdh*	*atp1a*
		1.32	1.68	2.45	4.61	4.68	5.18	7.00	8.00
	Gills	*h3a*	*actb*	*eif3g*	*ef1a*	*18s*	*gapdh*	*tuba*	*atp1a*
		1.00	2.91	3.16	3.46	3.50	5.42	7.00	8.00
	Hepatopancreas	*h3a*	*actb*	*gapdh*	*18s*	*eif3g*	*ef1a*	*tuba*	*atp1a*
		1.50	2.21	2.71	3.46	4.40	4.43	7.00	8.00
	Kidney	*h3a*	*actb*	*eif3g*	*ef1a*	*18s*	*gapdh*	*tuba*	*atp1a*
		1.57	2.45	2.63	3.16	3.60	5.48	6.74	8.00

In the Roundup® group the most stable gene across different ages was *h3a* in adults and immature and *ef1a* for juveniles. In different tissues, the most stable gene was also *h3a* in gills and hepatopancreas, but *actb* was most stable in brain and kidney. As expected, the gene with the lowest stability was *atp1a* across different ages and tissues, except in hepatopancreas, where *tuba* exhibited the lowest stability (Table 4).

**Table 4:** Consensus stability ranking by RefFinder of the candidate reference genes in *Odontesthes humensis* exposed to Roundup®.

**Roundup® exposure (10 mg.L**^**−1**^**, a.e.)**		**Ranking**							
		**1**	**2**	**3**	**4**	**5**	**6**	**7**	**8**
Ages	Adults	*h3a*	*eif3g*	*actb*	*18s*	*ef1a*	*tuba*	*gapdh*	*atp1a*
		1.57	2.00	2.28	3.46	4.68	5.23	7.00	8.00
	Juveniles	*ef1a*	*actb*	*18s*	*h3a*	*eif3g*	*gapdh*	*tuba*	*atp1a*
		1.41	1.57	2.78	3.66	4.95	5.86	6.48	8.00
	Immatures	*h3a*	*actb*	*ef1a*	*18s*	*eif3g*	*tuba*	*gapdh*	*atp1a*
		1.19	2.21	2.71	3.34	4.23	6.24	6.74	8.00
Tissues	Brain	*actb*	*eif3g*	*18s*	*h3a*	*tuba*	*ef1a*	*gapdh*	*atp1a*
		1.32	1.68	3.98	4.16	4.28	4.61	5.86	8.00
	Gills	*h3a*	*tuba*	*eif3g*	*actb*	*18s*	*ef1a*	*gapdh*	*atp1a*
		1.41	2.66	2.78	3.46	4.30	5.18	5.24	8.00
	Hepatopancreas	*h3a*	*actb*	*eif3g*	*18s*	*gapdh*	*ef1a*	*atp1a*	*tuba*
		1.32	2.21	2.82	3.13	4.95	5.48	6.73	7.24
	Kidney	*actb*	*h3a*	*eif3g*	*gapdh*	*ef1a*	*18s*	*tuba*	*atp1a*
		1.68	1.86	2.45	3.50	4.16	6.09	6.24	8.00

The *h3a* gene displayed the highest stability in different developmental stages in the seawater group. It was also the most stably expressed gene in the gills and hepatopancreas, while *actb* was the most stable in brain and kidney. The gene with the lowest stability across the ages and tissues was *atp1a*, except in hepatopancreas, where *tuba* exhibited the lowest stability in expression (Table 5).

**Table 5:** Consensus stability ranking by RefFinder of the candidate reference genes in *Odontesthes humensis* exposed to seawater.

**Seawater exposure (30 ppt)**		**Ranking**							
		**1**	**2**	**3**	**4**	**5**	**6**	**7**	**8**
Ages	Adults	*h3a*	*eif3g*	*18s*	*actb*	*ef1a*	*gapdh*	*tuba*	*atp1a*
		1.57	2.00	2.59	3.64	4.43	5.66	6.45	7.11
	Juveniles	*h3a*	*ef1a*	*eif3g*	*18s*	*actb*	*gapdh*	*tuba*	*atp1a*
		1.19	2.00	3.00	4.30	4.43	5.23	6.16	7.74
	Immatures	*h3a*	*eif3g*	*ef1a*	*18s*	*actb*	*tuba*	*gapdh*	*atp1a*
		1.57	2.28	2.83	3.16	3.16	6.00	7.00	8.00
Tissues	Brain	*actb*	*h3a*	*eif3g*	*gapdh*	*ef1a*	*18s*	*tuba*	*atp1a*
		1.19	1.41	3.22	4.23	5.23	5.66	6.24	8.00
	Gills	*h3a*	*ef1a*	*actb*	*eif3g*	*18s*	*tuba*	*gapdh*	*atp1a*
		1.32	2.63	2.91	2.99	4.30	5.23	6.24	8.00
	Hepatopancreas	*h3a*	*actb*	*eif3g*	*gapdh*	*18s*	*ef1a*	*atp1a*	*tuba*
		1.68	2.00	2.59	4.12	4.30	5.44	6.26	6.45
	Kidney	*h3a*	*eif3g*	*actb*	*18s*	*gapdh*	*ef1a*	*tuba*	*atp1a*
		1.32	1.68	3.22	3.34	4.23	6.24	6.74	8.00

**Figure 6:**
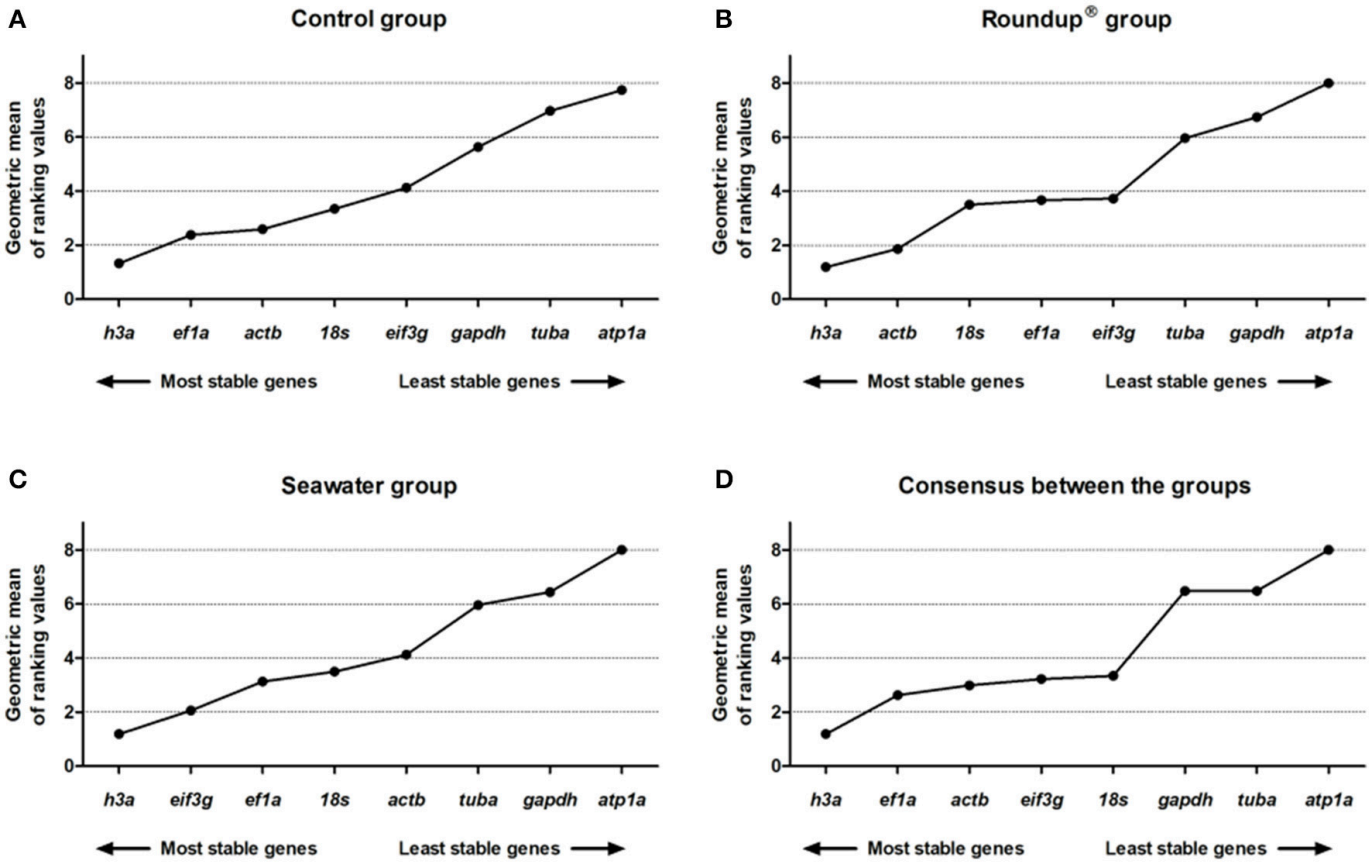
Consensus stability analysis of the candidates reference genes in *Odontesthes humensis* calculated by RefFinder algorithm. Gene expression stability in silversides exposed to water with quality parameters controlled **(A)**; to Roundup® Transorb [10 mg.L^−1^ (acid equivalent) of glyphosate] **(B)**; and to seawater (30 ppt) **(C)** across the different treatments for 24 h; and final consensus stability analysis between all methods and grouping the three treatments groups **(D)**. The most stable genes are displayed on the left, and the least stable genes are displayed on the right of the x-axis.

In the control group, the consensus comprehensive ranking constructed by RefFinder was *h3a* (1.32) > *ef1a* (2.38) > *actb* (2.59) > *18s* (3.34) > *eif3g* (4.12) > *gapdh* (5.63) > *tuba* (6.96) > *atp1a* (7.74) (Figure 6A). In the Roundup® group, the ranking order of stability was *h3a* (1.19) > *actb* (1.86) > *18s* (3.50) > *ef1a* (3.66) > *eif3g* (3.72) > *tuba* (5.96) > *gapdh* (6.74) > *atp1a* (8.00) (Figure 6B). The consensus ranking of stability constructed based on the expression data of the seawater group was: *h3a* (1.19) > *eif3g* (2.06) > *ef1a* (3.13) > *18s* (3.50) > *actb* (4.12) > *tuba* (5.96) > *gapdh* (6.44) > *atp1a* (8.00) (Figure 6C). The final consensus stability analysis, considering all methods and grouping the different tissues, ages, and treatments groups, revealed the following ranking order: *h3a* (1.19) > *ef1a* (2.63) > *actb* (2.99) > *eif3g* (3.22) > *18s* (3.34) > *gapdh* (6.48) > *tuba* (6.48) > *atp1a* (8.00) (Figure 6D).

## Discussion

In the present study, it was used individual methods (comparative dCt, geNorm, NormFinder, and BestKeeper) to obtain independent rankings of gene expression stability of candidate RGs in *O. humensis* and an algorithm that deduces a consensus ranking among all methods (RefFinder) such as presented in recent studies about RGs (Taki et al., 2014; Xu et al., 2014; Huang et al., 2015). Different algorithms can give different results depending of the set of analyzed genes and experimental variables (Yang et al., 2013; Zhang et al., 2013; Liu et al., 2014; Purohit et al., 2016) and no one statistical approach can cover all variables (Taki et al., 2014). For these reasons, the use of more than one algorithm is indicated. The different results presented by each algorithm are natural and expected due to their distinct statistical approach to construct the rankings (Liu et al., 2014). For example, the dCt method compares the relative expression of gene pairs with their SD values to get the most stable. GeNorm goes beyond, it calculates the *M-*value from the SD values and the geometric mean, and compares all gene pairs results. BestKeeper also starts from the SD values of gene expression and a geometric mean of the genes with the most stable Ct value are used to calculate the BestKeeper index. Then, it is calculated the Pearson's correlation coefficient ([r]) and the *P*-value to determine the similarity between the candidate RGs expression. The NormFinder is based in analysis of variance (ANOVA) and it is the unique of the used algorithms that evaluates the intragroup variation (i.e., replicates of a same treatment group) besides the intergroup variation (i.e., control vs. Roundup® or seawater groups), which also used by the other pairwise comparisons approaches, and combine both variations into a stability value. In this study, some differences were obtained in the ranking orders occupied by the genes in result of each algorithm analysis mainly from the second to the penultimate positions (Figures 2–5). Due to this, the use of the analysis by the ReFinder, which calculates the geometric mean for the previously obtained ranks, was important to a consensual unification of the discrepant results.

In the current study, the most stable gene across the treatment groups, independent of the method of analysis, including the final consensus RefFinder analysis, was *h3a*. This gene was also the most stable across different ages, except in the juveniles from the Roundup® group. However, in the consensus analysis of the tissues samples revealed *h3a* to be not as stable as in the age analysis. For example, the results of brain analysis demonstrated this gene not to be the most stable in all treatments. Moreover, *h3a* did not exhibit the highest stability in the kidney of Roundup® group. The *h3a* gene has already been evaluated in studies for validation of RGs showing high stability (Taylor et al., 2007; Koenigstein et al., 2013) and can be used to normalize qRT-PCR reactions (Ramachandra et al., 2008). However, up to now, no study has reported this gene as the candidate RG in a fish species. In the final consensus ranking, the second most stable gene was *ef1a*. All performed analysis revealed this gene to have high stability, occupying at least the third position of the constructed rankings, except in consensus ranking of the Roundup® group constructed by RefFinder, where it occupied the fourth position. A deviation in the ranking order of stability is natural owing to the use of different algorithms. Studies on stability of candidates RGs in different experimental protocols have revealed that *ef1a* is usually the most stable gene of fishes (Jorgensen et al., 2006; Tang et al., 2007, 2012; Infante et al., 2008; Liman et al., 2013; Hu et al., 2014).

The studies that evaluated the gene expression in the South American silversides have used *actb* as the internal control for qRT-PCR (Strobl-Mazzulla et al., 2005; Karube et al., 2007; Fernandino et al., 2008, 2011; Majhi et al., 2009; Miranda et al., 2009; Blasco et al., 2010; Gómez-Requeni et al., 2012; Pérez et al., 2012; González et al., 2015). In the present study, RefFinder analysis focused on the individual tissues revealing *actb* to be the most stable gene in brain and kidney from the Roundup® group and in the brain from the seawater group. Only the geNorm analysis of stability, based on control and Roundup® groups, revealed *actb* as the most stable gene. However, our results showed that *actb* was not the most stable gene. The RefFinder final consensus comprehensive ranking pointed this gene as the third most stable in *O. humensis*. There is still much debate on the use of *actb* to normalize qRT-PCR reactions in fishes. Some authors have reported that this gene is the least stable in some fish species (Jorgensen et al., 2006; Filby and Tyler, 2007; Yang et al., 2013; Hu et al., 2014; Chapman and Waldenström, 2015). However, the studies that present *actb* as a good RG have also reported that its high stability in fishes is tissue dependent within a species (Zheng and Sun, 2011; Zhang et al., 2013; Sun and Hu, 2015), such as in *O. humensis*. This does not mean that the studies using *actb* as the RG should not be considered. However, future studies should pay more attention to the choice of RG.

The *18s* gene has been a subject of discussions on its use as the RG in fish models. This gene has been shown as the most or to be among the most stable candidates in some fish species (Jorgensen et al., 2006; Filby and Tyler, 2007; Tang et al., 2007; Small et al., 2008; Yang et al., 2013; Liu et al., 2014). However, in other species, this gene is considered unsuitable for use as an internal control for qRT-PCR (Infante et al., 2008; Hu et al., 2014; Purohit et al., 2016). In *O. humensis* from control, Roundup®, and seawater groups, and across different ages and tissues, *18s* generally held the intermediary position in the rankings, attracting little attention for its use in normalization methods. The *gapdh* gene, as well as *actb* and *18s*, has presented discrepant results in the literature that reports its expression stability. The *gapdh* has already been reported as one of the most stable genes in half-smooth tongue sole (Liu et al., 2014). Furthermore, its expression is usually stable depending on the evaluated tissue, being able to occupy both extremities of rankings of stability (Yang et al., 2013; Zhang et al., 2013). So, generally, *gapdh* is refuted as a suitable gene for an efficient normalization (Jorgensen et al., 2006; Filby and Tyler, 2007; Tang et al., 2007, 2012; Infante et al., 2008; Small et al., 2008; Ahi et al., 2013; Liman et al., 2013; Hu et al., 2014; Chapman and Waldenström, 2015). In the present study, *gapdh* appeared to be the second most unstable gene, only after *atp1a* gene.

The *tuba* gene usually presents high stability in studies on validation of candidates RGs. This gene was the second most stable after analysis by dCt and NormFinder in Nile tilapia under normal conditions (Yang et al., 2013). This gene also exhibited high stability in intestine and liver from Japanese flounder at 24 and 72 h after viral infection, respectively, as well as in muscle of turbot after 72 h post-infection (Zhang et al., 2013). Even with *tuba* gene presenting higher stability in the brain of silversides from the control group, this gene did not maintain this expression pattern. In the brain of silversides from other treatment groups, *tuba* had low stability levels. Across other tissues and different developmental stages, the *tuba* gene frequently occupied the last places of the final ranking, even behind *atp1a*, as well as across treatment groups and different algorithms used. The final consensus comprehensive ranking constructed by RefFinder presented *tuba* as the most unstable gene in *O. humensis*, disregarding *atp1a*. Finally, the control gene *atp1a*, despite being a constitutive gene, was the most unstable, as expected. It occupied the last position in the rankings of stability constructed by all algorithms used to compare the three treatment groups. This gene also exhibited lower stability across different ages and tissues from silversides from all treatments, except in the hepatopancreas of the Roundup® and seawater groups.

In the present study, a set of candidates of RGs was analyzed for the first time in *O. humensis*. The genes *atp1a, tuba, gapdh*, and *18s* were the least stable and highly unsuitable to be used for qRT-PCR normalization in studies in silverside *O. humensis*. The three most stable genes were *h3a, ef1a*, and *actb*. The discordance observed among the results of analyses of the candidate RGs in different fish species, tissues, developmental stages, and experimental conditions, it is evident the importance of identification of efficient RGs for each experimental design instead of the indiscriminate use of traditional genes. Furthermore, the gene *h3a* exhibited a greater potential to be used as a RG in *O. humensis* in comparison with other traditional genes, such as *actb* and *ef1a*. Perhaps the *h3a* gene had the same potential to be used as the best RG in other fish species, since the stability of this gene has never been evaluated in other fishes. Therefore, future studies warrant the evaluation of *h3a* as candidate RG in other fish species, besides *O. humensis* or silversides.

## Author contributions

TS and VC are responsible for experimental design, data analysis, manuscript writing. TS, MR, and RR were responsible for fish acclimation and maintenance. TS, BB, IL, LS, MR, RR, and WD were responsible for the biological collections. TS, BB, IL, LS, WD, and VC were responsible for the molecular biology, from RNA extraction to sequencing and qRT-PCR analysis. TC and FS were also responsible for qRT-PCR analysis. AV, CC, and DM were responsible for the flow-cytometry analysis.

### Conflict of interest statement

The authors declare that the research was conducted in the absence of any commercial or financial relationships that could be construed as a potential conflict of interest.

## Abbreviations

RG: reference gene.
