# Supplementary material for: Evaluation of Reference Genes to Analyze Gene Expression in Silverside Odontesthes humensis Under Different Environmental Conditions
Source: Front Genet. 2018 Mar 14;9:75. doi: 10.3389/fgene.2018.00075 (PMC5861154; doi:10.3389/fgene.2018.00075)
Supplement: Supplementary file 1 [file Table1.docx]

**Supplementary table 1.** Mean RNA integrity number (RIN) ± standard error mean (SEM) of tissue samples collected from *Odontesthes humensis* exposed to the experimental groups: control, Roundup^®^ Transorb [10mg.L^−1^ (acid equivalent, a.e.) of glyphosate], and seawater (30 ppt).

| **Tissues** | **Treatments** | **RIN ± SEM (N = 3)** |
| --- | --- | --- |
| Brain | Control | 8.5 ± 0.3756 |
|  | Roundup®  (10 mg.L^-1^, a.e.) | 8.3 ± 0.1528 |
|  | Seawater  (30 ppt) | 8.3 ± 0.2646 |
| Gills | Control | 8.5 ± 0.2186 |
|  | Roundup®  (10 mg.L^-1^, a.e.) | 8.2 ± 0.1202 |
|  | Seawater (30 ppt) | 8.6 ± 0.3383 |
| Hepatopancreas | Control | 8.5 ± 0.3215 |
|  | Roundup®  (10 mg.L^-1^, a.e.) | 8.3 ± 0.5132 |
|  | Seawater (30 ppt) | 8.0 ± 0.1202 |
| Kidney | Control | 8.4 ± 0.5508 |
|  | Roundup®  (10 mg.L^-1^, a.e.) | 9.1 ± 0.1202 |
|  | Seawater (30 ppt) | 8.8 ± 0.3606 |
