# Supplementary material for: Evaluation of Reference Genes to Analyze Gene Expression in Silverside Odontesthes humensis Under Different Environmental Conditions
Source: Front Genet. 2018 Mar 14;9:75. doi: 10.3389/fgene.2018.00075 (PMC5861154; doi:10.3389/fgene.2018.00075)
Supplement: Supplementary file 2 [file Table2.docx]

**Supplementary table 2.** Species and respective sequences (with accession numbers) used to obtain the initial primers, through alignment and selection of conserved regions, for cloning and sequencing of reference genes in *Odontesthes humensis*.

| **Gene symbol** | **Gene name** | **Species** | **GenBank accession No.** |
| --- | --- | --- | --- |
| *18s* | 18S ribosomal RNA | *Bidyanus welchi* | HQ615530 |
|  |  | *Arripis truttaceus* | HQ615529 |
|  |  | *Salmo salar* | FJ710886 |
| *actb* | β-Actin | *Odontesthes bonariensis* | EF044319 |
| *ef1a* | Elongation factor 1-α | *Paralichthys olivaceus* | AB915949 |
|  |  | *Epinephelus coioides* | HQ441076 |
|  |  | *Danio rerio* | L23807 |
| *eif3g* | Eukaryotic translation initiation factor 3g | *Sciaenops ocellatus* | JX002675 |
|  |  | *Gasterosteus aculeatus* | BT028147 |
|  |  | *Tetraodon nigroviridis* | CR723985 |
|  |  | *Salmo salar* | BT049926 |
| *gapdh* | Glyceraldehyde-3-phosphate dehydrogenase | *Cyprinus carpio* | JX244278 |
|  |  | *Danio rerio* | AY818347 |
|  |  | *Coregonus clupeaformis* | KP893543 |
| *h3a* | Histone h3a | *Ctenopoma acutirostre* | AY662878 |
|  |  | *Oreochromis mossambicus* | AY662948 |
|  |  | *Epinephelus itajara* | EF120885 |
| *atp1a* | Na^+^/K^+^-ATPase-α | *Monopterus albus* | KC620449 |
|  |  | *Solea senegalensis* | AB759894 |
|  |  | *Scatophagus argus* | KF649219 |
|  |  | *Dicentrarchus labrax* | KP400258 |
|  |  | *Siganus canaliculatus* | EU107280 |
| *tuba* | Tubulin-α | *Chionodraco rastrospinosus* | AF263276 |
|  |  | *Salmo salar* | BT043911 |
|  |  | *Oreochromis mossambicus* | FN673693 |
